# Comparison of Auditory Perception Scores Following Unilateral Cochlear Implantation in Children From Different Regions Using Neubio’s Full-Spectrum Continuous Stimulation

**DOI:** 10.7759/cureus.76247

**Published:** 2024-12-23

**Authors:** Santosha C D, Jitendra Mohan Hans, Rahul Agrawal, Shashank Vashist

**Affiliations:** 1 Business and Management, Christ University, Bengaluru, IND; 2 Department of Ear, Nose, and Throat (ENT), Dr. Hans Centre for Hearing and Vertigo Clinic, New Delhi, IND; 3 Department of Ear, Nose, and Throat (ENT), Cochlear Implant Unit, Agrawal Hospital and Research Institute, Gwalior, IND; 4 Department of Otolaryngology, Manipal Hospital, Gurugram, IND

**Keywords:** auditory verbal habilitation (avh), cochlear implantation (ci), likert rating scale, meaningful auditory integration scale, northern india, revised cap, urban vs rural

## Abstract

Aims and objectives: The study aimed to compare the auditory perception status of children from different socioeconomic backgrounds, specifically urban versus rural. It also examined the correlation between outcome measures and the frequency of auditory verbal therapy sessions attended, as well as the impact of continuous electric analog stimulation on the age of implantation.

Material and methods: A retrospective cohort study was carried out on 30 children who have received unilateral cochlear implantation in rural versus urban backgrounds. Revised category of auditory perception (CAP) scores and meaningful auditory-integration scale (MAIS) were compared at set time frequencies of pre-switch on, three months, six months, and nine months post-implantation between the two groups of implanted children.

Results: Revised CAP and MAIS scores showed improvement over time with auditory verbal therapy. Both groups of implantees showed better improvement irrespective of the background. The children implanted more than five years of age showed a significant improvement over time with continuous electric analog signals.

Conclusion: Intensive rehabilitation is essential for children who have received cochlear implantation in which their required needs are addressed individually and optimized for the best outcomes. The study shows that hearing from the implant can be improved and have meaning with the help of auditory verbal therapy for optimal frequency irrespective of the background to a notable extent.

## Introduction

For children with severe to profound hearing loss, a cochlear implant can be a lifesaver in terms of both hearing and communicating [1]. Recent developments in cochlear implantation technology have resulted in significant enhancements in postoperative results [2]. There is still a group of children that do not receive the maximum benefit, even after using the device every day for several years [3]. One of the elements that influence the results of cochlear implantation is the ethnic heritage of the implanted children, which can be broadly categorized as either urban or rural backgrounds. Urban and rural areas are phrases used to categorize areas with human habitation. The primary distinctions between urban and rural places lie in their population density and level of development, although there are additional factors to consider [4].

According to Peng et al. cochlear implantation, along with auditory rehabilitation, leads to an improved ability to perceive sound and understand spoken language [5]. According to Mahone and Schneider, auditory verbal treatment results in a greater level of improvement in auditory resolution and speech perception [6]. Hence, it is recommended that parents ensure their child participates in auditory verbal therapy sessions for a duration of up to two years following the implantation. Parents must possess significant motivation and obedience in order to meet the tight schedules of therapy sessions.

Few research findings suggested that children from higher socioeconomic backgrounds make more progress in the areas of spoken language and comprehension, whereas no significant differences were observed in the revised category of auditory perception (CAP) and meaningful auditory-integration scale (MAIS) scores [7]. In the Indian context, there is ample literature stating the difficulties of children from rural backgrounds in attaining auditory and speech outcomes post-cochlear implantation [8]. The full-spectrum continuous stimulation (FSCS) coding strategy converts a digital signal to an electric analog signal and sends a full-spectrum coded signal to the internal receiving unit (IRU) at a continuous rate. Manufacturers code the signal in such a way that speech becomes a priority, and the patient can hear speech clearly [9].

The ultimate goal for prelingual pediatric patients is to achieve not only hearing but also clear and effective speech. To assess auditory performance, various tools and scales are utilized. These include the revised CAP score, which offers a hierarchical evaluation of auditory perception, ranging from basic awareness of environmental sounds to complex tasks such as telephonic conversations [10]. Furthermore, the MAIS evaluates, via interviews with parents, how children with significant hearing loss make use of sound in their daily lives.

In this study, we aim to delve into the multifaceted impact of cochlear implantation and therapy attendance on auditory outcomes among children in age groups ranging from two to 16 years. Understanding these complex dynamics is pivotal for optimizing rehabilitation strategies and ensuring that all children, regardless of their backgrounds, have equitable access to the benefits of cochlear implantation.

This research underwrites the broader field of audiology and rehabilitation by shedding light on the interplay of therapy attendance, socioeconomic factors, and cochlear implant outcomes in children, with particular emphasis on the role of FSCS in affecting auditory performance. The insights gleaned from this study can form healthcare policies and interventions aimed at improving one's standard of living and means of expression for children with hearing impairments.

## Materials and methods

This study was a retrospective observational comparison audit that assessed the validated rehabilitation ratings of 30 children with severe to profound hearing loss, who did not have any other problems, and who underwent cochlear implantation with the BOLD 22 CI system. The cochlear implantation employs the manufacturer, Neubio's “Full-Spectrum Continuous Stimulation (FSCS)’’ coding technique. The strategy rather than delivering pulses of localized stimulation like other manufacturers provides continuous stimulation of a sinusoidal wave to the spiral ganglion for a full spectrum signal. By sending the entire frequency range through the electrode array where the resistance of each successive electrode provides a natural fall, off of energy distinguished into high and low frequencies from base to the apex, on the basilar membrane, a large electric field is created, which covers the entire neural spiral ganglia and further allows it to naturally encode that information for further processing in the brain. The input signals first split into 32 frequency bands in the digital sound processor to manipulate dynamic range, noise levels, and current. The processor then codes this signal continuously in the time domain and preserves frequencies to create a close-to-natural input sound wave. This sound wave is then superimposed on a carrier wave and transmitted to the implant. The study data using the FSCS coding strategy were retrieved from patients' records from December 2021 until October 2022.

Children aged two to 16 years receiving unilateral cochlear implants were studied, and divided into urban and rural groups based on their living area's population, with urban areas defined by the 2020 census criteria of at least 2,000 housing units or a population over 5,000. Population density was determined using Google searches based on addresses from the records. The study, conducted in Cochlear Implant Clinics in Delhi and Gwalior, tracked variables like parental attendance at speech therapy sessions over three, six, and nine months post-implant activation, linking these to the children's auditory outcome improvements. Data included demographics, implant details, and pre- and post-implantation auditory scores (revised CAP and MAIS) at specified intervals, including an analysis of late implanters to assess the effects of continuous electric analog signals on auditory outcomes.

Participants

Children implanted between the ages of two and 16 years at the time of implantation, with bilateral congenital severe to profound sensorineural hearing loss, normal intellect, and radiologically normal results were included (Tables 1, 2).

**Table 1:** Participants age distribution in the study Table 1 presents an inclusive overview of the demographic characteristics of the participants involved in the study.

Age range	No. of male	Mean age	Standard deviation	No. of females	Mean age	Standard deviation
2- 5 yrs	07	4.14	+/- 0.98	13	3.77	+/- 1.3
6-10 yrs	03	6	0	04	8.75	+/- 1.96
11-16 yrs	-	-	-	03	14	+/- 1.63

**Table 2:** Participant's time duration completed in the study Table 2 outlines the duration of participation post-cochlear implant device activation. It shows that 12 children completed nine months, 18 children completed six months, and 30 children completed three months.

S.no	Duration completed post-switch on	Mean age	Standard deviation	No. of patients
1.	3 months	06	+/-3.19	30
2.	6 months	6.05	+/-3.28	18
3.	9 months	7.14	+/-4.57	12

Pre- and post-analysis

Pre- and post-analysis is a methodological approach used in experimental studies to measure the effect of an intervention by comparing data collected before (pre-) and after (post-) the intervention. In this context, the intervention typically refers to a treatment, procedure, or any other form of experimental manipulation.

Pre-analysis

Pre-analysis involves gathering baseline data before the experimental intervention is introduced. In the study, this would include assessing the children’s auditory perception and related capabilities before the activation of their hearing implants. The purpose of pre-analysis is to establish a reference point against which post-intervention outcomes can be compared. This baseline data is crucial because it 1) establishes the initial condition: It provides a clear picture of the children's auditory abilities before receiving the hearing implants, ensuring that any changes observed afterward can be attributed to the intervention, and 2) identifies variability: by understanding the baseline differences among participants, the study can account for individual variability, which helps in more accurately interpreting the results.

Post-analysis

Post-analysis is conducted after the intervention has taken place. In this study, post-analysis involves assessing the children’s auditory perception at several intervals (e.g., three, six, and nine months) following the activation of their hearing implants. The post-analysis serves several important purposes. 

Measures the effectiveness of the intervention: By comparing post-intervention data with the pre-intervention baseline, the study can determine the degree of improvement in auditory perception due to the hearing implants.

Tracks progress over time: Conducting post-analysis at multiple intervals allows the study to observe how the benefits of the intervention develop or change over time, providing insights into both the short-term and long-term effectiveness of the treatment.

Rationale for using pre- and post-analysis

The study employs pre- and post-analysis to ensure a robust experimental design that can accurately evaluate the impact of the hearing implants on children's auditory abilities. This approach is used because of the following reasons: 1) controls for confounding variables: By using each participant as their own control (comparing their post-intervention data to their pre-intervention baseline), the study minimizes the impact of external factors that could otherwise influence the results; 2) enhances validity: The comparison between pre- and post-data strengthens the study's internal validity, providing stronger evidence that observed changes are due to the intervention itself; and 3) enables detailed outcome measurement: Pre- and post-analysis allows for a detailed understanding of the intervention’s effects, offering insights into the magnitude, timing, and consistency of improvements in auditory perception. Overall, pre- and post-analysis are essential for determining the efficacy of hearing implants and understanding how they influence auditory development over time.

Assessment of pre- and post-operative measures

Revised Categories of Auditory Perception

The revised CAP scores were utilized to quantify the level of auditory perception. The scale is a numerical range from 0 to 12. A score of 0 means that the child has no awareness of surrounding sounds, while a score of 12 indicates that the youngster is capable of conversing on the phone with an unfamiliar speaker. This choice was made because it provides a more practical assessment of auditory performance and was measured before and after three, six, and nine months of the switch-on.

Meaningful Auditory Integration Scale

The MAIS is a questionnaire that parents complete through an interview-style format. The scoring method comprises 10 questions, with each question being assigned a rating ranging from 0 to 4 points. The score with the least value is denoted as 0, while the score with the greatest value is denoted as 4. The cumulative score is calculated by summing the points earned in each of the 10 questions. The MAIS assesses the child's capacity to link sounds with occurrences in their surroundings, without necessitating the demonstration of word recognition abilities. The scale was selected to evaluate children who speak different languages in order to obtain data that can be compared. It was used to examine the progress before and after three, six, and nine months of the switch on.

Likert Rating Scale

The five-point frequency Likert scale was used to measure the frequency of therapy attendance by children. It is a scale with points ranging from 1 to 5, wherein a score of 1 represents "never," 2 - "rarely," 3 - "sometime," 4 - "often," and 5 - "always."

The scores were determined by the auditory-verbal therapist who was in charge of the education and treatment of the youngsters. The MAIS questionnaires were administered to auditory verbal therapists, who recorded the replies to each question in the corresponding patient's case file. The revised CAP, MAIS, and Likert rating scores of all 30 children, collected at three, six, and nine months after switching on their hearing devices, were analyzed for correlation with the parents' frequency of attending speech therapy, as scored on a Likert scale. The therapy's frequency was assessed based on the number of sessions attended throughout the specified time period.

Statistical analysis

The data was evaluated using a standardized statistical program (SPSS v20.0, Vermont, USA) with the assistance of a biostatistician. The scores obtained before and after the implantation procedure were compared and analyzed for correlation at specific time intervals. A Mann-Whitney test was employed to compare the two groups of individuals who received implants over a period of time. This test also examined whether there were any variations in results based on the age at which the implantation occurred, using a continuous electric analog signal. The Friedman test and Pairwise Wilcoxon signed rank test were conducted to assess the differences in scores over the time scale. A Spearman correlation test was used to assess the relationship between the outcome measures and the frequency of therapy on the Likert scale in children residing in rural and urban areas.

## Results

A total of 30 bilateral severe to profound hearing loss children who have received unilateral cochlear implantation from urban and rural backgrounds were included in this study. Here, in the findings, extensive light is shed on the utilization of cochlear implants with the FSCS coding strategy on the auditory performance of children, with a focus on various key variables such as comparison based upon the region (urban vs. rural), age stratification (less than five years vs. more than five years), and therapy frequency (three, six, and nine months) (Table 3).

**Table 3:** Post-switch on auditory performance scores analysis by months and region *Significant at P<0.05 **Significant at P<0.01

Months	Post-switch on	N	Region	Minimum	Maximum	Mean	SD	Median	Mean rank	|Z|	p-value
3 months	Revised CAP	15	Urban	1	6	3.60	1.45	3.00	18.90	2.152	0.031^*^
		15	Rural	0	5	2.33	1.45	2.00	12.10		
	MAIS	15	Urban	3	36	19.13	10.29	18.00	16.47	0.603	0.546
		15	Rural	6	31	16.47	7.77	15.00	14.53		
	Likert scale	15	Urban	2	5	3.47	0.99	4.00	17.23	1.126	0.260
		15	Rural	2	5	3.07	0.96	3.00	13.77		
6 months	Revised CAP	9	Urban	3	7	4.22	1.48	4.00	10.94	1.187	0.235
		9	Rural	1	6	3.33	1.94	3.00	8.06		
	MAIS	9	Urban	16	39	26.67	8.02	30.00	10.83	1.061	0.289
		9	Rural	5	32	20.78	11.22	26.00	8.17		
	Likert scale	9	Urban	2	5	3.44	1.01	4.00	10.28	0.644	0.520
		9	Rural	1	5	3.11	1.17	3.00	8.72		
9 months	Revised CAP	7	Urban	3	8	5.00	1.83	5.00	6.86	0.423	0.673
		5	Rural	3	7	4.60	2.19	3.00	6.00		
	MAIS	7	Urban	15	36	27.29	7.99	28.00	6.86	0.409	0.683
		5	Rural	9	36	24.40	11.41	26.00	6.00		
	Likert scale	7	Urban	3	5	3.71	0.95	3.00	7.00	0.602	0.547
		5	Rural	2	5	3.40	1.52	3.00	5.80		

Comparative analysis of auditory outcomes in urban and rural areas

Descriptive analysis and Mann-Whitney test evaluated revised CAP and MAIS scores along with Likert scale ratings at three, six, and nine months post-cochlear implant activation. Initially, a significant difference in revised CAP scores was observed between urban (mean=3.60) and rural (mean=2.33) regions at three months. However, no significant differences were found in revised CAP scores at six and nine months, nor in MAIS scores at these later time points in either region, despite overall improvements indicating enhanced outcomes post-implantation.

The MAIS mean scores were 19.13 in three months which increased to 27.29 after nine months in the urban region, and in the rural region, the MAIS mean increased from 16.47 to 24.40, indicating a gradual improvement over time. The Likert scale measure showed results, with no significant changes in the mean scores over time. Urban region consistently had higher mean scores than the rural region, with a mean score of 3.71 compared to 3.40 after nine months but it was not statistically significant to find the difference (Table 4).

**Table 4:** Descriptive statistics for auditory performance measures and Likert scale of frequency of therapy at different time intervals *Significant at P<0.05 **Significant at P<0.01

Variables	Tests	Minimum	Maximum	Mean	SD	Median	Mean rank	χ^2^(3)	p-value
Revised CAP	Pre-switch on	0	3	0.17	0.59	0.00	1.04	32.510	0.000^**^
	After 3 months	0	6	2.97	1.56	3.00	2.17		
	After 6 months	1	7	3.78	1.73	3.00	3.21		
	After 9 months	3	8	4.83	1.90	4.50	3.58		
MAIS	Pre-switch on	0	11	0.37	2.01	0.00	1.00	26.415	0.000^**^
	After 3 months	3	36	17.80	9.06	15.50	2.38		
	After 6 months	5	39	23.72	9.93	27.00	3.21		
	After 9 months	9	36	26.08	9.19	27.00	3.42		
Likert scale	After 3 months	2	5	3.27	0.98	3.00	1.75	1.600	0.449
	After 6 months	1	5	3.28	1.07	3.00	2.17		
	After 9 months	2	5	3.58	1.16	3.00	2.08		

The test shows a significant difference in the scores comparing the data from the pre-switch on values. The minimum and maximum values, mean, standard deviation, median, mean rank, χ2(3), and p-value are reported for each test at different times. The results show that for the pre-switch on the revised CAP score, the minimum and maximum values were 0 and 3, while the minimum increased to 3 and the maximum to 8 after nine months. The revised CAP mean score increases from 0.17 at pre-switch to 4.83 after nine months. Similarly, for the MAIS score, the minimum value increases from 0 to 9, and the maximum value remains between 11 and 36, while the mean score increases from 0.37 to 26.08 after nine months. The results also show that there is a significant difference in the mean rank and χ2(3) values between the pre-switch on and Post-three months, and pre-switch on and post-six months tests for both revised CAP and MAIS tests (p<0.01). However, for the Likert scale test, there is no significant difference (p>0.05) between the values reported at different times (Table 5).

**Table 5:** Comparative analysis of pre- and post-auditory performance measures at different time intervals *Significant at P<0.05 **Significant at P<0.01

Parameters	Pairs											
	Pre- vs. post-3 months		Pre- vs. post-6 months		Pre- vs. post-9 months		3-month vs. 6-month		3-month vs. 9-month		6-month vs. 9-month	
	|Z|	p-value	|Z|	p-value	|Z|	p-value	|Z|	p-value	|Z|	p-value	|Z|	p-value
Revised CAP	4.731	0.000^**^	3.755	0.000^**^	3.086	0.002^**^	2.057	0.040^*^	2.842	0.004^**^	1.826	0.068
MAIS	4.786	0.000^**^	3.725	0.000^**^	3.061	0.002^**^	2.550	0.011^*^	2.847	0.004^**^	0.356	0.722

Table 5 shows the results of comparing various pairs of parameters including pre- vs. post-three, six, and nine months, three-month vs. six-month, three-month vs. nine-month, and six-month vs. nine-month for revised CAP and MAIS. The results are presented in terms of Z scores and p-values. For revised CAP, all comparisons show a significant difference with p-values <0.05, and the majority of them with p-values <0.01. For MAIS, all comparisons show a significant difference with p-values <0.05, with the exception of the six-month vs. nine-month comparison, which is not significant.

Age stratification (less than five years vs. more than five years)

By categorizing participants into two age groups who have completed their nine months, the study examined how age impacted auditory performance after cochlear implantation, offering valuable age-specific insights (Table 6).

**Table 6:** Comparative analysis of auditory performance measures stratified by age groups and time intervals *Significant at P<0.05 **Significant at P<0.01

Months	Variables	Groups	n	Minimum	Maximum	Mean	SD	Median	Mean rank	Mann-Whitney test	
										|Z|	p-value
3 month	Revised CAP	Less than 5 years	5	1	3	2.33	0.82	2.50	4.92	2.235	0.025^*^
		More than 5 years	7	1	6	4.00	1.50	4.00	10.06		
	MAIS	Less than 5 years	5	6	31	19.00	8.17	18.50	8.50	0.355	0.723
		More than 5 years	7	6	32	17.78	8.66	15.00	7.67		
6 month	Revised CAP	Less than 5 years	5	2	6	3.50	1.38	3.00	6.08	1.411	0.158
		More than 5 years	7	3	7	4.56	1.51	4.00	9.28		
	MAIS	Less than 5 years	5	10	32	24.33	8.41	27.00	7.08	0.650	0.516
		More than 5 years	7	17	39	27.78	7.21	30.00	8.61		
9 month	Revised CAP	Less than 5 years	5	3	7	4.00	1.55	3.50	5.92	1.510	0.131
		More than 5 years	7	3	8	5.56	1.94	6.00	9.39		
	MAIS	Less than 5 years	5	13	34	23.50	7.61	24.50	6.75	0.888	0.375
		More than 5 years	7	9	36	27.22	10.53	32.00	8.83		

Table 6 compares implantation effects in two age groups, <5 years (n=5) and >5 years (n=7), analyzing revised CAP and MAIS scores at three, six, and nine months post-implant. Initially, at three months, the <5 years group showed a significantly lower revised CAP mean (2.33 vs. 4.00, p=0.025) but no MAIS difference. At six months, both groups had similar revised CAP scores (3.50 vs. 4.56) and MAIS scores (<5 years: 24.33, >5 years: 27.78, p=0.516). By nine months, though no significant differences were noted, the <5 years group had lower mean scores in both revised CAP (4.00 vs. 5.56, p=0.131) and MAIS (23.50 vs. 27.22, p=0.375).

The Friedman test indicated significant improvements in revised CAP and MAIS scores over time in a study of 12 cochlear implant recipients. These findings suggest that the participants' revised CAP scores improved significantly over time. For the MAIS variable, there was also a significant difference in mean rank across the three time points (χ2(2) =8.561, p<0.05), indicating changes in the MAIS scores over time. Pairwise comparisons showed that the mean rank for MAIS at three months was significantly lower than at six months (Z=1,40, p<0.01) and nine months (Z=2.33, p<0.05). These results suggest that the participants' MAIS scores improved significantly from three to six and nine months after implantation. Overall, these findings suggest that both the revised CAP and MAIS scores improved significantly over time after cochlear implantation in both of the age groups. However, the study's small sample size limits the generalization of the findings to a broader population (Tables 7, 8).

**Table 7:** Comparison of auditory performance measures (revised CAP and MAIS) at different time intervals for patients more than five years *Significant at P<0.05 **Significant at P<0.01 CAP, categories of auditory perception; MAIS, meaningful auditory-integration scale

Variables	Months	Minimum	Maximum	Mean	SD	Median	Mean rank	χ^2^(2)	p-value
Revised CAP	3 month	1	6	3.33	1.496	3.00	1.33	17.778	0.000^**^
	6 month	3	7	4.13	1.506	4.00	2.00		
	9 month	3	8	4.93	1.907	4.00	2.67		
MAIS	3 month	6	32	18.27	8.189	18.00	1.40	8.561	0.014^*^
	6 month	17	39	26.40	7.614	28.00	2.27		
	9 month	9	36	25.73	9.362	26.00	2.33		

**Table 8:** Comparison of auditory performance measures (revised CAP and MAIS) in patients with cochlear implanted less than five years of age over three time points *Significant at P<0.05 **Significant at P<0.01 CAP, categories of auditory perception; MAIS, meaningful auditory-integration scale

Variables	Months	Minimum	Maximum	Mean	SD	Median	Mean Rank	χ^2^(2)	p-value
Revised CAP	3 month	1	3	2.33	0.82	2.50	1.08	10.300	0.006^**^
	6 month	2	6	3.50	1.38	3.00	2.17		
	9 month	3	7	4.00	1.55	3.50	2.75		
MAIS	3 month	6	31	19.00	8.17	18.50	1.58	1.909	0.385
	6 month	10	32	24.33	8.41	27.00	2.08		
	9 month	13	34	23.50	7.61	24.50	2.33		

The Friedman test on recipients less than 5 years of age (n=5) that are implanted revealed significant improvements in revised CAP scores over time at three, six, and nine months post-implant activation (χ2(2)=10.300, p=0.006) but not in MAIS scores (χ2(2)=1.909, p=0.385). Revised CAP scores significantly rose from three to nine months, with notable increases between three and six months (p=0.034) and between three and nine months (p=0.024), but not between six and nine months (p=0.083). MAIS scores showed no significant changes at any time point. This suggests significant auditory perception improvements in revised CAP but not in MAIS for these patients (Table 9).

**Table 9:** Pairwise comparisons of auditory performance measures (revised CAP and MAIS) over time for patients with cochlear implanted more than five years of age *Significant at P<0.05 **Significant at P<0.01 CAP, categories of auditory perception; MAIS, meaningful auditory-integration scale

Variables	Pairs	|Z|	p-value
Revised CAP	3 month	0.686	0.493
	6 month		
	3 month	2.214	0.027^*^
	9 month		
	6 month	2.041	0.041^*^
	9 month		
MAIS	3 month	2.313	0.021^*^
	6 month		
	3 month	2.494	0.013^*^
	9 month		
	6 month	0.059	0.953
	9 month		

## Discussion

It is crucial to understand the implications of the FSCS coding strategy in a broader context, and its effect on the auditory perception outcomes in children from different regions. The study findings have important implications for our understanding of auditory perception in cochlear implant patients aged over five years. The results provide a comprehensive insight into the auditory perception of children who underwent cochlear implantation, with a focus on various key variables. These variables include region (urban vs. rural), which elucidates the influence of geographical factors on post-implantation progress, age stratification (less than five years vs. more than five years), offering age-specific insights into auditory performance, and therapy frequency (three, six, nine months), which assesses the effects of different therapy time frequencies post-device activation.

This study aims to evaluate how therapy frequency impacts cochlear implant outcomes in children from rural and urban backgrounds. Table 3 and Table 4, categorizing data by region, enhance our understanding of how diverse settings and ages affect auditory perception in children with implants. Table 4 shows both groups making significant progress in revised CAP scores three, six, and nine months post-activation, with urban children showing a notable early advantage at three months. However, this urban-rural gap in revised CAP scores closed over time, and MAIS scores improved consistently in both regions, indicating positive outcomes across the board. The detailed analysis underscores the crucial role of therapy frequency and suggests that both urban and rural children benefit substantially from cochlear implants, with variations in progress attributable to different factors, including initial baseline capabilities and intervention strategies.

Table 5 details statistical comparisons of auditory performance measures over time using Z-scores and p-values, shedding light on the significance of auditory perception improvements from cochlear implants. These analyses, along with those in Table 4 and Table 5, collectively underscore the beneficial effects of cochlear implants on children's hearing.

In this study, age was proven to be a significant variable as mentioned in Table 6 and Table 7 for implanted participants of more than five years of age; there was a statistically significant improvement in revised CAP scores over time, specifically from three to nine months post-switch on. This suggests that children aged more than five years benefited from cochlear implantation and experienced significant improvements in auditory perception abilities post-implantation. Table 7 analyzes participants as a whole, without dividing them into subgroups, aligning with previous data showing improved auditory perception post-cochlear implantation.

Following the acknowledgment of age as a significant factor in cochlear implantation outcomes, the frequency and duration of therapy also emerge as pivotal contributors to enhancing hearing skills among children over five years of age. As demonstrated in Table 9, within this subgroup (children over five years old), cochlear implantation led to substantial improvements in auditory perception abilities during the initial nine months post-implantation, underscoring the potential for significant enhancements in auditory perception within this timeframe. Cochlear implant recipients showed significant improvements in revised CAP scores over time, especially between three and six months and three and nine months post-switch on. Children implanted before age five initially had lower scores at three months but improved greatly, reaching scores similar to those implanted after age five by nine months. This may be due to younger children's shorter attention spans. Attention issues are common in preschoolers, with 40% of four-year-olds having notable attention problems [10]. These results highlight the importance of age and therapy duration in cochlear implant outcomes.

These findings emphasize the importance of considering age at implantation when evaluating outcomes and tailoring intervention strategies to address the unique needs of younger recipients, particularly in terms of fostering their attention skills alongside auditory development. Our study findings echo the importance of analog signaling in neural networks and cortical information transmission, as highlighted by Asfour et al. [11].

This highlights that while auditory skills may not exhibit significant age-related variations, auditory perception abilities can vary based on the age at which the implantation occurs [12].

Our study further corroborates the significance of utilizing continuous electric analog signals in cochlear implant stimulation [13]. This innovative approach holds substantial promise, especially for children undergoing cochlear implantation after the age of five. Our study's findings align with this research by García et al. demonstrating remarkable improvements in auditory outcomes among older children who received cochlear implants, emphasizing the practical benefits of this pioneering approach in the field [14].

This approach has the potential to enhance auditory outcomes development, addressing the challenges often faced by older pediatric implant recipients. By providing a full spectrum continuous signal, these implants align with the principles of neural plasticity, allowing the brain to adapt and reorganize in response to sensory input, as highlighted in various studies by Van et al. [15]. The concept of electrical stimulation is supported by research indicating that neuronal networks in the mammalian brain operate as analog devices, inspiring the creation of artificial neural networks that mimic analog models as suggested by Sladen et al. [16]. Analog axonal signaling, as demonstrated by Alle et al., significantly contributes to information transmission within local cortical networks [17].

One of the studies by Peters et al., intriguing outcomes, in line with is that socioeconomic status did not significantly influence children's performance, with both rural and urban backgrounds showing similar outcomes in terms of revised CAP and MAIS scores 7 [18]. This finding challenges the assumption that rural residence might impact a child's therapy attendance [19].

In summary, our research findings indicate that the use of FSCS electric analog signals in the cochlear implant can lead to notable improvements in auditory outcomes, even in older children who receive cochlear implants after the age of five. It is important to note that while older children may initially show faster progress within the first nine months, our study suggests that younger children have the potential to catch up and reach the same level as their hearing peers in the long term. This alignment between our research results and the application of analog signaling highlights the practical significance and effectiveness of this innovative approach in the field of cochlear implantation.

Limitations of the study

This study's limitations need to be acknowledged. First, the sample size is relatively small, with only 30 participants, which may limit the generalizability of the findings to a broader population of children with bilateral severe to profound hearing loss. Additionally, the study only included children who received unilateral cochlear implants, and the results may not apply to those who received bilateral implants. Another limitation is the lack of long-term follow-up beyond nine months, which restricts the ability to assess the sustained effectiveness of the FSCS coding strategy on auditory performance over time. Furthermore, the study does not account for other variables that may influence auditory outcomes, such as the type of therapy received, the quality of care, or socioeconomic factors beyond the urban-rural classification. Finally, the study relies on self-reported measures and parental assessments, which may introduce bias or inaccuracies in reporting.

## Conclusions

This study has shown that cochlear implantation in children from rural vs. urban backgrounds has provided benefits in auditory perception. The use of FSCS electric analog signal for cochlear implant stimulation can be an innovative and noteworthy technique within the realm of cochlear implants in children more than five years of age of implantation. Intensive auditory verbal Habilitation is essential for children who have received cochlear implantation, especially for children irrespective of the background for the best outcomes.
